# Efficacy and safety of Xinmai’an tablet in treatment of premature ventricular contractions due to coronary heart disease: A protocol for systematic review and meta-analysis

**DOI:** 10.1097/MD.0000000000032253

**Published:** 2022-12-09

**Authors:** Meiling Wang, Sihua Che, Weiwei Pan, Shumao Zhang, Guijun Shi

**Affiliations:** a School of Traditional Chinese Medicine, Changchun University of Traditional Chinese Medicine, China; b Changchun Medical College, China; c Rehabilitation Hospital of Jilin Province Disabled People’s Federation, China; d Changchun Chinese Medicine Hospital, China.

**Keywords:** coronary heart disease, premature ventricular contractions, Xinmai’an tablet

## Abstract

Coronary heart disease (CHD) is the leading cause of human death in the world and a public health problem of global concern. As a common arrhythmia in CHD, premature ventricular contractions are very likely to progress to fatal arrhythmias, resulting in serious adverse consequences. At present, the treatment of premature ventricular contractions due to CHD mainly focuses on catheter ablation, beta-blockers and antiarrhythmics. Both require ongoing monitoring because relapses may lead to redevelopment of cardiomyopathy, and there are varying degrees of indications and side effects. Several clinical studies have shown that Xinmai’an can effectively control the occurrence of premature ventricular contractions and reduce the risk of recurrence. However, there is currently no systematic review evaluating its effectiveness. Therefore, the purpose of this study is to provide strong evidence-based medical evidence for Xinmai’an tablet in the treatment of premature ventricular contractions due to CHD.

**Methods:**

We will search the main Chinese and English databases from inception to June 5, 2022. And identified as the randomized controlled trials. In addition, a reference list of studies meeting the inclusion criteria will be retrieved. Two researchers will conduct literature screening and quality evaluation. And we will conduct bias risk assessment and sensitivity analysis. The analysis software uses RevMan 5.3.

**Results:**

Mainly by observing the number of ventricular premature beat attacks (24-hour holter monitoring electrocardiogram), electrocardiogram efficacy (ST segment and T wave changes) and echocardiogram assesses the structure and function of the left and right ventricular, left ventricular ejection fraction, etc. To evaluate the clinical effect of Xinmai’an on premature ventricular contractions due to CHD.

**Conclusion:**

The results of this study will provide a basis for the selection of treatment options for premature ventricular contractions due to CHD.

## 1. Introduction

Coronary heart disease (CHD) is a cardiovascular disease caused by coronary atherosclerosis that leads to stenosis or occlusion of the official cavity. CHD and its complications are the main causes of death worldwide, and the mortality rate is closely related to the degree of coronary artery involvement and myocardial dysfunction.^[1,2]^ Premature ventricular contractions are the most common arrhythmia in CHD. Frequent premature ventricular contractions can aggravate the condition of patients with CHD over time and become a trigger for fatal arrhythmias, such as ventricular tachycardia and ventricular fibrillation, resulting in sudden cardiac death.^[3,4]^ It seriously affects the quality of life and health of contemporary human beings. At present, the clinical treatment of this disease mainly includes catheter ablation, beta-blockers and antiarrhythmic drugs. Although the curative effect is accurate, but there are different degrees of indications and side effects.^[5]^ Catheter ablation is an invasive treatment that may result in varying degrees of complications such as arteriovenous fistula or inguinal hematoma, cardiac perforation with tamponade perforation, intraoperative stroke or death.^[6]^ There are age and indication limitations, and there is a risk of postoperative infection and coronary artery injury.^[7]^ Drug therapy such as bisoprolol, atenolol, amiodarone, etc, its long-term use is limited by its adverse effect, which is not conducive to prognosis.^[8,9]^ Randomized trials have shown that antiarrhythmic drugs can suppress premature ventricular contractions, but they also increase the risk of CHD death.^[10]^ However, traditional Chinese medicine (TCM) has the advantage of adjusting the overall function of patients and long-term application without obvious adverse reactions in the treatment of ventricular premature contractions, and is also significantly better than anti-arrhythmic Western drugs in the long-term efficacy of drugs and improving the quality of life of patients.^[11]^

Xinmai’an tablet is a Chinese patent medicine composed of ginseng, milkvetch root, radix salviae miltiorrhizae, peony root, dwarf lilyturf tuber, and borneol, which has the effect of tonifying qi, nourishing yin, activating blood, and regulating palpitation.^[12]^ Modern pharmacological studies have found that Xinmai’an tablet has the effects of dilating coronary artery, increasing coronary artery flow, slowing heart rhythm and reducing myocardial contractility, which can significantly reduce the total incidence of ventricular arrhythmia caused by myocardial ischemia in rats.^[13]^ Among them, the active ingredient ginsenoside Re (G-Re) in ginseng has antiarrhythmic effect. In addition, G-Re also exerts antiischemic effect and induces angiogenic regeneration.^[14]^ Clinical studies have shown that Xinmai’an tablet has achieved significant curative effects in the treatment of this disease, and has few side effects, suitable for long-term use, and can also play a role in consolidating the efficacy and reducing recurrence.^[15,16]^ However, the evidence of Xinmai’an tablet in treating premature ventricular contractions due to CHD has not been systematically evaluated. In this study, we will evaluate the clinical efficacy of randomized trials by rigorous systematic evaluation and meta-analysis.

## 2. Methods

### 2.1. Protocol and registration

This protocol will be implemented under the Preferred Reporting Projects for Systematic Reviews and Meta-Analyses Protocols (PRISMA-P) guidelines.^[17]^ Furthermore, the program has been registered with PROSPERO (registration number: CRD42022353958).

### 2.2. Type of study

This study will include literature on clinical randomized controlled trials (RCTs) of Xinmai’an tablet in the treatment of premature ventricular contractions duo to CHD, as well as search reference lists of studies that met the inclusion criteria. No language, publication, time or blinding restrictions are involved.

### 2.3. Type of participant

The selection of patients will meet the diagnostic criteria of CHD and premature ventricular contractions.^[18]^ The grading of premature ventricular contractions will use the Lown Woff grading method. There are no limitations on race, sex, and age.

### 2.4. Intervention

The control group was given conventional treatment of western medicine for basic diseases, such as aspirin enteric-coated tablets, statins, angiotensin converting enzyme inhibitor, and other drugs, with no limit on specific drugs, dosages and methods, and on the basis of routine treatment, beta-blocker and antiarrhythmic Western drug was given, such as metoprolol tartrate and bisoprolol, etc, were used in accordance with the recommended dosage of instructions; the observation group was given Xinmai’an tablet orally on the basis of routine western medicine treatment.

### 2.5. Outcomes

The primary outcomes will include the number of ventricular premature beat attacks (24-hour holter monitoring electrocardiogram), electrocardiogram efficacy (ST segment and T wave changes) and echocardiogram assesses the structure and function of the left and right ventricular, left ventricular ejection fraction and clinical effective rate. The secondary outcomes will be adverse events, 6-minute walk test (6MWT), efficacy of clinical symptoms (disappearance or significant improvement/improvement/no change in symptoms such as chest distress and palpitations) and TCM syndrome score scale were measured before and after treatment.^[19]^

### 2.6. Exclusion criteria

1. The subjects of the study are diseases other than premature ventricular contractions due to CHD (such as sick sinus syndrome, ventricular fibrillation, atrial flutter, etc)
2. Literature with no primary outcome measure or unpublished and inaccessible literature.

### 2.7. Search strategy

The database search will include PubMed, Embase, Cochrane Library, Web of Science, Wanfang, China national knowledge infrastructure, Chinese biomedical database, and Chinese Scientific Journal Database database through June 5, 2022. And identified as the RCTs. In addition, a reference list of studies meeting the inclusion criteria will be retrieved. Using the following words to search, such as “Xinmai’an tablet,” “premature ventricular contractions,” “CHD,” etc. Seeing Table 1 for details.

**Table 1:** Search strategy of the PubMed.

Number	Search terms
#1	Coronary Heart Disease[Mesh]
#2	Coronary Heart[Title/Abstract] OR Heart Diseases[Title/Abstract] Atherosclerosis Diseases[Title/Abstract] OR Cardiovascular Diseases[Title/Abstract] Coronary[Title/Abstract] OR Coronary Artery Disease[Title/Abstract]
#3	#1 AND #2
#4	Premature Ventricular Contractions[Mesh]
#5	Ventricular premature beat[Title/Abstract] OR Ventricular extrasystole[Title/Abstract] OR ventricular ectopic beat[Title/Abstract]
#6	#4 AND #5
#7	#3 AND #6
#8	Xinmai’an tablet[Mesh]
#9	Xinmai’an[Title/Abstract] OR Xinmai’an tablets[Title/Abstract] OR Chinese patent medicine[Title/Abstract]
#10	#8 AND #9 OR #7 AND #8 OR #7 AND #9 OR #8 AND #3 AND #6
#11	Controlled trial [Publication Type] OR Randomized [Publication Type] OR Controlled clinical trial[Publication Type] OR Randomized Controlled trial[Title/Abstract] OR protocol[Title/Abstract]
#12	#3 AND #11 OR #6 AND #11 OR #10 AND #11

### 2.8. Data collection and analysis

#### 2.8..1. Selection of literatures

**Figure 1.**
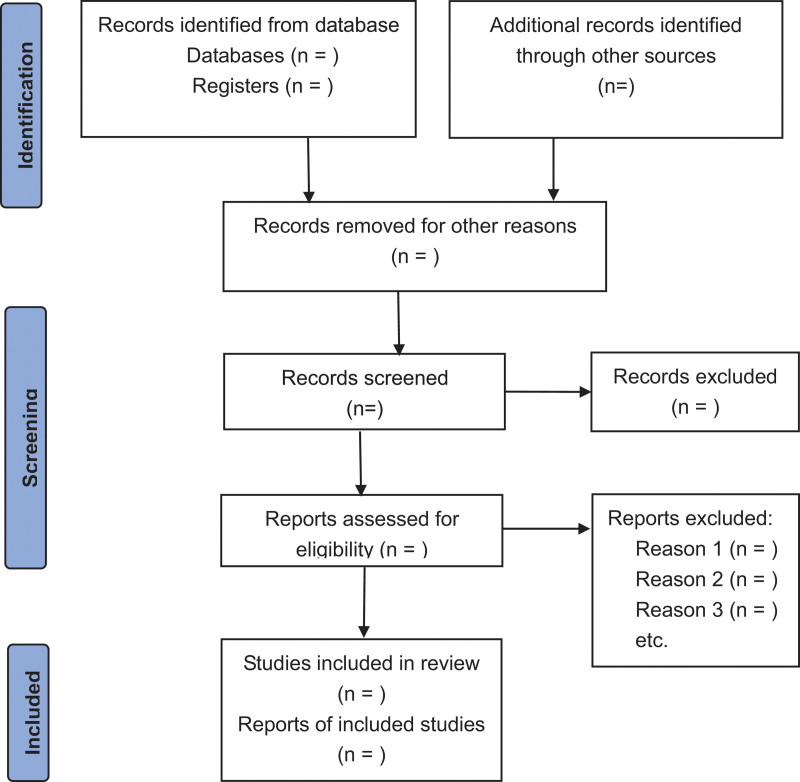
PRISMA flow diagram of the study selection process.

First, the literature will be searched and screened, and the qualified literature will be imported into EndnoteX8 software. Among them, 2 researchers (Meiling Wang and Sihua Che) will sort out the collected literature, delete unqualified and duplicated literature, and be responsible for recording. Further review is then carried out by downloading the full text. Finally, articles that met the inclusion criteria were selected, cross-checked by 2 researchers and extracted relevant data. In case of disagreement, a third researcher (Panwewe) will assist in mediation. The specific flow chart is shown in Figure 1 according to the PRISMA principle.

#### 2.8..2. Data extraction and management

Two authors (Meiling Wang and Shumao Zhang) will use Excel2013 tool to extract data from the included studies, including author’s name, publication year, title, study characteristics (cohort, clinical case-control study or RCT, etc), study population (number of participants and distribution of age, sex, and race), intervention methods, outcomes and adverse events, etc. If important information is missing from the inclusion process, we will attempt to contact the authors for more complete and comprehensive information. If there is any objection, the third author (Guijun Shi) will decide whether to implement it or not.

#### 2.8..3. Risk of bias assessment

Risk of bias in this study will be independently assessed by 2 investigators (Meiling Wang and Sihua Che), using the Cochrane tool.^[20]^ We will set “low risk,” “high risk,” and “unclear risk” as the risk levels for this assessment, which mainly include random sequence generation, methods of concealing treatment allocation, as well as whether bias sources such as trial participants, medical providers, outcome assessors, data integrity, analysis and data collectors are blind, and if there are differences in the selection of outcome data, a senior third investigator (Weiwei Pan) would coordinate and resolve them.

#### 2.8..4. Data synthesis strategy

In this study, the RevMan5.0 software (version 5.3) provided by Cochrane Company will be used for data synthesis. Mean difference or standard mean difference with 95% confidence interval will be used for pooled effect measures to calculate continuous data. Dichotomous outcomes will be measured by the rate ratio or odds ratio with 95% confidence intervals. If there is no significant heterogeneity among studies (*I*^*2*^ < 50%, *P* > .1), the fixed effect model can be used. When significant heterogeneity occurs among the studies (*I*^*2*^ ≥ 50%, *P* < .1), a random-effect model is performed to analyze data. We will perform subgroup analysis and sensitivity analysis in order to identify factors affecting heterogeneity. We will conduct Begger’s funnel plots and Egger’s linear regression tests to investigate the publication bias.

#### 2.8..5. Assessment of heterogeneity and sensitivity analysis

When there is heterogeneity among the results of the various studies, a multi-faceted subgroup analysis such as CHD type, age, gender, and duration of treatment will be performed. Sensitivity analysis maintains stability of results by eliminating low-quality studies and performing profiling again.

#### 2.8..6. Publication bias

If >10 patients will be included in eligible studies, funnel plots will be used to test for bias and Egger’s test will be used for symmetric analysis.

#### 2.8..7. Grading the quality of evidence

The quality of the evidence will be divided into 4 grades, namely high, moderate, low and very low quality, and the whole study will be assessed for the reliability of the evidence, assign and evaluate the grades.^[21]^

#### 2.8..8. Ethics and dissemination

This protocol is a systematic review and meta-analysis study, which does not contain private information of patients, so it does not require the approval of the ethics committee, and the results of this study will be published in a peer-reviewed journal.

## 3. Discussions

As we all know, CHD has a high diagnosis rate and mortality rate, which is a worldwide problem. With the acceleration of aging population, premature ventricular contractions due to CHD have received more and more attention, and with the continuous development of TCM theory, its long-term efficacy has also been better affirmed. It belongs to the category of palpitation in TCM. Its clinical manifestations are chest tightness, fatigue, shortness of breath and tremor cordis.^[22]^ Although Western medicine has a certain efficacy in the treatment of premature ventricular contractions due to CHD, there are more adverse reactions when taken for a long time. However, in the treatment mode of integration of traditional and western medicine, TCM can alleviate the adverse reactions of Western medicine, so that the efficacy is fast and lasting, and at the same time, when Western medicine treats the primary disease, TCM can play an auxiliary effect and improve the symptoms of ventricular premature beats. The 2 methods can play a complementary and mutually reinforcing role, and the treatment of premature ventricular contractions has more advantages and better application prospect.^[23]^ So we will provide evidence-based medicine evidence for clinical treatment of premature ventricular contractions by collecting available evidence for systematic analysis.

## Author contributions

**Conceptualization:** Meiling Wang.

**Data curation:** Sihua Che, Weiwei Pan.

**Funding acquisition:** Guijun Shi.

**Investigation:** Shumao Zhang.

**Methodology:** Meiling Wang.

**Project administration:** Guijun Shi.

**Resources:** Weiwei Pan.

**Supervision:** Shumao Zhang.

**Validation:** Sihua Che.

**Writing – original draft:** Meiling Wang.

**Writing – review & editing:** Meiling Wang.
